# Editorial: Translational Approaches for Targeting Cardiovascular Complications of Diabetes

**DOI:** 10.3389/fphar.2021.799020

**Published:** 2021-11-17

**Authors:** Arpeeta Sharma, Miles J. De Blasio, Mitchel Tate, Rebecca H. Ritchie, Andrew J. Murray

**Affiliations:** ^1^ Oxidative Stress Laboratory, Baker Heart and Diabetes Institute, Melbourne, VIC, Australia; ^2^ Drug Discovery Biology, Monash Institute of Pharmaceutical Sciences, Parkville, VIC, Australia; ^3^ Department of Pharmacology, Monash University, Melbourne, VIC, Australia; ^4^ Department of Pharmacology and Therapeutics, The University of Melbourne, Melbourne, VIC, Australia; ^5^ Department of Physiology, Development and Neuroscience, University of Cambridge, Cambridge, United Kingdom

**Keywords:** cardiovascular disease, diabetes, novel therapeutic strategies, mechanisms of diseases, preclinical models

The global prevalence of diabetes has increased over several decades, and by 2045 approximately 700 million individuals will be affected, representing a global epidemic and significant socioeconomic burden (Saeedi et al., 2019). Diabetes is a major risk factor for complications including nephropathy, neuropathy, retinopathy, and cardiovascular disease (CVD) (Gilbert et al., 2006). In addition, diabetes can have a direct impact on the heart increasing the risk of heart failure (HF) (Kannel et al., 1974), a condition termed diabetic cardiomyopathy (DbCM). The risk of developing HF is 5-8-fold higher in 45–65 year old patients with diabetes compared with non-diabetic individuals (Gilbert et al., 2006). DbCM is a complex disease manifesting as maladaptive changes in cardiac structure and function independent of other risk factors such as hypertension, coronary artery disease, and atherosclerosis (Rubler et al., 1972; Ritchie and Abel, 2020). Features of DbCM include left ventricular diastolic and systolic dysfunction, concomitant with cardiac oxidative stress, inflammation, mitochondrial abnormalities and remodeling including cardiomyocyte hypertrophy, interstitial fibrosis, and apoptosis (Tate et al., 2017; Marwick et al., 2018; Tate et al., 2019; De Blasio et al., 2020; Makrecka-Kuka et al., 2020). Current research is focused on factors and pathways that drive DbCM, to elucidate potential therapeutic approaches.

Fibrosis is a key driver for the development of diastolic dysfunction and cardiomyopathy in the setting of diabetes. The bone morphogenetic proteins (BMPs) are members of the transforming growth factor-beta (TGF-β) superfamily, and mediate diverse effects at the cellular level. While TGF-β initiates a pro-fibrotic response, BMP7 appears to counter this, and may in fact be antifibrotic. Tate et al. used a cardiac-directed adeno-associated viral (AAV) vector-mediated gene therapy approach to explore the therapeutic potential of BMP7 in a mouse model of type 1 diabetes (T1D) with impaired diastolic function. Their findings include reductions in cardiomyocyte hypertrophy and apoptosis, decreased cardiac fibrosis and improved LV function. Strikingly, these improvements were seen following a single administration of the rAAV6-BMP7 vector. Further work is needed to elucidate mechanisms underpinning this protection, but the authors highlight the promise of this approach, either as a standalone therapy or in combination with existing strategies.

Inflammation is a further mechanism implicated in many chronic diseases. In their review article, Henson and Aksentijevic consider the inflammatory landscape of the diabetic heart, and the emergence of immunosenescence as a possible driver of cardiac dysfunction. The authors discuss how senescent cell load accumulates in cardiovascular diseases, including HF, and posit that systemic metabolic stress in the context of type 2 diabetes (T2D) might result in the development and accumulation of senescent cells. The authors highlight interventional studies that aim to target ageing, inflammation and related cellular phenotypes in CVD and T2D. The review aims to spark a wider discussion and generate hypotheses linking senescence to cardiac dysfunction in the context of diabetes. The authors highlight unanswered questions and areas where further research is needed, including in the crosstalk between immune cells and senescent cells of the diabetic heart.

Cardiac arrhythmias are commonly seen in the setting of DbCM. A review article by Veitch et al., explores emerging evidence for calcium/calmodulin-dependent kinase II (CaMKII) as a therapeutic target in DbCM. The authors consider mechanisms that might underpin increased CaMKII activation in the diabetic heart, including oxidation and O-GlcNAcylation; modifications which the authors suggest might represent effective therapeutic targets. The article summarises the major targets of CaMKII, and downstream routes to cardiac dysfunction including arrhythmogenesis and alterations in contractility and excitation-contraction coupling. The authors propose that CaMKII inhibition could be an appealing avenue for therapy, representing a single target that might allow for the treatment of a host of downstream components of diabetes-related cardiac dysfunction.

The vascular complications of diabetes are typically characterised by endothelial dysfunction and abnormal angiogenesis. There are however no current treatments to stimulate angiogenesis. MicroRNA (miRNA)-based therapies, albeit in their infancy, are emerging as powerful tools to treat complex diseases as they can target multiple genes and regulate the translational expression of multiple proteins. Solly et al. review the potential role of miR-181c as a therapeutic target for rescuing diabetes-impaired angiogenesis. The authors describe the implications of impaired angiogenesis in diabetic vascular complications, in particular wound healing and ischaemia, and highlight how miR-181c is involved in key cellular processes and signalling pathways that mediate classical angiogenic responses, including cellular proliferation and survival, invasion and migration, and mitochondrial function as well as tissue remodelling. The authors suggest that targeting miR-181c may be beneficial for orchestrating the multiple factors required for adequate stimulation of angiogenesis in a clinical setting.

Diabetes is also an independent risk factor for atherosclerotic peripheral artery disease (PAD), and consequences can include lower limb claudication and, in severe cases, amputation. Stimulation of angiogenesis is a potential therapeutic strategy, and Bubb et al. explored the use of CL 316,243, a β_3_ adrenergic receptor (β_3_AR) agonist, to promote this response on the basis of previous findings that β_3_AR agonism improved nitric oxide (NO)/redox balance. After demonstrating the effectiveness of this approach in established cell-based assays of angiogenesis, the researchers imposed hind limb ischaemia on mouse models of T1D and T2D, finding that CL 316,243 increased angiogenesis and improved recovery, in a NO-dependent manner.

In population-based studies, high dietary fructose has been associated with altered expression of mitochondrial, apoptotic and oxidative stress-related proteins, leading to the accumulation of cardiac lipid species and hence diastolic dysfunction, an effect that is substantially increased in the setting of diabetes. Annandale et al. highlight the implications of fructose stress on the pathogenesis of DbCM and the need for further interventional studies that specifically target fructose-related pathways in the heart, an important therapeutic avenue in mitigating diabetic heart disease.

Amino acids play critical roles in gene expression, metabolism, oxidative stress and inflammation in the heart and other organs, with disturbances in amino acid metabolism linked to CVD. Alqudah et al. report an observational study, comparing plasma amino acids in Jordanian subjects with T2D and non-diabetic counterparts. These data were obtained in relatively lean individuals whose T2D was treated with single glucose-lowering therapy (metformin), which was not optimally-controlled (fasting blood glucose and glycated hemoglobin levels remained elevated). Although there were no differences in total, non-essential or semi-essential amino acids between the groups, there were differences in levels of individual amino acids, including marked elevation of plasma aspartate and reduced plasma serine in metformin-treated T2D individuals. These differences at the level of individual amino acids may inform future studies identifying therapeutic targets of glucose-lowering therapies, perhaps as a biomarker for effectiveness over the longer-term.

Despite progress in understanding this disease process, there are currently no effective treatments that specifically target the underlying pathogenesis contributing to DbCM (Marwick et al., 2018). Understanding the complexities of the factors involved in the development of DbCM could improve diagnosis, allowing for earlier detection, and ultimately lead to more effective treatment strategies.
